# Exploring networks of care in implementing midwife-led birthing centres in low- and middle-income countries: A scoping review

**DOI:** 10.1371/journal.pgph.0001936

**Published:** 2023-05-23

**Authors:** Sabera Turkmani, Andrea Nove, Oliva Bazirete, Kirsty Hughes, Sally Pairman, Emily Callander, Vanessa Scarf, Mandy Forrester, Shree Mandke, Caroline S. E. Homer

**Affiliations:** 1 Burnet Institute, Melbourne, Victoria, Australia; 2 University of Technology Sydney, Sydney, Australia; 3 Novametrics Ltd, Duffield, United Kingdom; 4 University of Rwanda, Kigali, Rwanda; 5 International Confederation of Midwives, The Hague, Netherlands; 6 Monash University, Melbourne, Victoria, Australia; American University of Beirut, LEBANON

## Abstract

The evidence for the benefits of midwifery has grown over the past two decades and midwife-led birthing centres have been established in many countries. Midwife-led care can only make a sustained and large-scale contribution to improved maternal and newborn health outcomes if it is an integral part of the health care system but there are challenges to the establishment and operation of midwife-led birthing centres. A network of care (NOC) is a way of understanding the connections within a catchment area or region to ensure that service provision is effective and efficient. This review aims to evaluate whether a NOC framework—in light of the literature about midwife-led birthing centres—can be used to map the challenges, barriers and enablers with a focus on low-to-middle income countries. We searched nine academic databases and located 40 relevant studies published between January 2012 and February 2022. Information about the enablers and challenges to midwife-led birthing centres was mapped and analysed against a NOC framework. The analysis was based on the four domains of the NOC: 1) agreement and enabling environment, 2) operational standards, 3) quality, efficiency, and responsibility, 4) learning and adaptation, which together are thought to reflect the characteristics of an effective NOC.Of the 40 studies, half (n = 20) were from Brazil and South Africa. The others covered an additional 10 countries. The analysis showed that midwife-led birthing centres can provide high-quality care when the following NOC elements are in place: a positive policy environment, purposeful arrangements which ensure services are responsive to users’ needs, an effective referral system to enable collaboration across different levels of health service and a competent workforce committed to a midwifery philosophy of care. Challenges to an effective NOC include lack of supportive policies, leadership, inter-facility and interprofessional collaboration and insufficient financing. The NOC framework can be a useful approach to identify the key areas of collaboration required for effective consultation and referral, to address the specific local needs of women and their families and identify areas for improvement in health services. The NOC framework could be used in the design and implementation of new midwife-led birthing centres.

## Introduction

Low and middle-income countries (LMICs) account for vast majority of all global maternal deaths. Most of these deaths could be prevented, with adequate resources, interventions, and an enabling environment. Poverty and geographical inaccessibility are key barriers to receiving safe and high-quality maternity services [1]. The evidence for the safety and benefits of midwifery services has been growing over the past two decades with recommendations that midwife-led care be scaled up in many countries [2, 3].

Many countries have implemented midwife-led care by establishing birthing centres. These centres can be situated within, or next to, a hospital, or can be freestanding [4]. There is evidence from high-income contexts that midwife-led birthing centres (MLBCs) are a safe birth setting for healthy pregnant women when midwives are well educated and adequate resources and infrastructure are available [5]. A key element of a safe and effective MLBC is well-functioning collaboration across service delivery with effective consultation and referral networks so that if women or newborns do develop complications, easy access to the next level of care is available. This approach is known as a Network of Care (NOC) [6].

A NOC refers to the intentional interconnections and communications between different levels of the health care system—including different professional disciplines or the public and private facilities—to improve health outcomes. Within each NOC there are common elements such as an enabling environment, a specific philosophy of being client centred, operational standards, policies, and interventions, all of which go towards ensuring quality and equity of care [6–9]. For example, in the provision of midwifery services, a NOC would enable a birthing centre to have collaborative and multi-disciplinary care that fulfils the needs of women [10]. For women, maternity care does not occur in isolation, rather women access multiple health care providers as a part of routine health care or management of pre-existing health conditions. A NOC can align health services, which are generally fragmented and poorly connected, to the health care needs of women. A NOC would also provide an approach to coordinate and manage service delivery, adapt to new challenges, and ensure the effective use of policies and resources [11]. NOCs also provide an opportunity to learn from actions, improve national policies, financing and governance and scale up health service delivery [10].

There are a few examples of how a NOC framework has assisted LMICs to refine or improve health services. In one example from Madagascar, the framework was used to discuss elements of collaboration for universal health coverage [8]. In Nigeria, the framework was used to address the specific local needs of women and their families and ensure they have access to timely and safe maternal and neonatal health services [9]. Similarly, in Tanzania, the framework was used to address gaps in maternal and newborn health service delivery [12] and also in Nepal, to describe the effects of biosocial interventions in transforming maternal and newborn health services in rural areas [7]. We were unable to find an example of using a NOC framework to analyse the strengths and weaknesses of MLBCs in LMICs.

This aim of this review therefore was to evaluate whether the NOC framework—in light of the literature about midwife-led birthing centres—can be used to map the challenges, barriers and enablers with a focus on low-to-middle income countries.

## Methods

This scoping review is part of a more extensive literature review on MLBCs in LMICs that provides evidence about the locations and characteristics of MLBCs in LMICs.

The search strategy included electronic searches from 9 online databases (CINAHL, Cochrane Library, EMBASE, LILASE, MEDLINE, PubMed, Sabinet, Scopus, Web of Science) combined with comprehensive secondary manual searches (S1 Table). Original research articles as well as case studies, reports and opinion pieces were considered. We used key terms such as “midwife-led”, “birth centre”, “birthing centre”, “normal birth centre”, “natural birth centre”, “midwifery unit”, or “midwifery clinic”. We included articles, that were about MLBCs in LMICs, care provided or led by midwives or nurse-midwives either within or outside of a health facility, care included but not limited to childbirth. Table 1 provides the inclusion and exclusion criteria.

**Table 1:** Inclusion and exclusion criteria.

Inclusion criteria	Exclusion criteria
Health facilities providing childbirth care in low- to middle-income countries as defined by the World Bank	Health facilities providing other types of health care, or health facilities providing childbirth care in high-income countries as defined by the World Bank.
Midwives or nurse-midwives are the lead professionals for childbirth care (whether a single midwife working alone, in a small team of midwives, a caseload model, or within an interdisciplinary team)	Midwives provide care under the leadership or direction of a doctor or other health professional, or midwives are the lead professional only by default (i.e. the midwife is the only available professional but there is no obvious commitment to the philosophy of midwife-led care), or the lead professional is a nurse without formal midwifery training, an auxiliary or associate midwife,^2^ a community health worker, or a traditional birth attendant
Childbirth care is provided in a dedicated (midwife-led) space either within or outside of a health facility^1^	Care is provided in another type of space within a health facility (e.g. a maternity ward or obstetric unit) or outside of a health facility (e.g. at the birthing woman’s home)
Item is a research study, report of activities, opinion piece, or conference abstract	Item is a review of the literature
Year of publication was 2012 or later	Year of publication before 2012
Published in English, French or Spanish	Published in other languages

^1^We did not exclude facilities if they did not fully meet the ICM working definition of an MLBC, because one of our aims was to identify and describe their characteristics, rather than to assume that the working definition applies in all contexts.

^2^ We were particularly interested in the role of midwives or nurse-midwives who usually have similar levels of education and generally can take leadership or higher level roles. Associate-level cadres usually have less formal education or training and were not the focus of this study.

This scoping review drew on the Carmone et al. 2020 [6] NOC framework, which defines characteristics of NOC. The framework is categorised in four domains: 1) agreement and enabling environment, 2) operational standards, 3) quality, efficiency and responsibility, 4) learning and adaptation, which together are thought to reflect the characteristics of an effective NOC. These four domains have subdomains that address issues such as quality of care, financing, community buy in, referral systems, supply and resources of health services to ensure services are reachable by those most in need [10] (Table 2).

**Table 2:** Networks of care domains and subdomains and their definitions.

Domain	Subdomain	Definition
**I: Agreement & enabling environment**	Policy	Mandate and decision-making power
	Financing	Affordability of services for the user, and appropriate budgeting for continued network of care operation
	Purposeful arrangements	What makes for effective network of care and differentiates them from a set of sites that have not invested effort in purposeful arrangements among them
	Buy-in and trusting relationships	Pertains to stakeholder engagement, professional culture, relationships with communities to be served
**II: Operational standards**	Referrals	Standards and systems for communication, transport and processes in complex cases
	Monitoring	Data recording, reporting and use to identify weaknesses
	Supply and infrastructure	Effective procurement and supply chain management for drugs, equipment, water and power
	Workforce	Efficient mix of workers, all skilled within their scope of practice, well distributed in adequate numbers
**III: Quality, efficiency, responsibility**	Coordination of care	Sense of shared agenda and shared responsibility
	Clinical guidance, documentation and review	Clear guidance for workers and robust data illustrating how the guidance is being applied—so they can “do the right thing, at the right time, by the right person”
	Benchmarking: skills, measurement and improvement	Systems for measuring and maximising clinical competence and quality of care
**IV: Learning and adaptation**	Client-centredness	Recognition that needs and preferences vary—increasing access for more vulnerable groups
	Flexibility; extending reach	Tendency to be flexible and attempt new approaches to extend reach, especially when providing services for hard-to-reach groups
	Evolution & resilience	The introduction of new approaches and technologies is a principal way that NOC evolve

Source: Adapted from Carmone et al 2020 [6]

Our definition of an MLBC was: “*a dedicated space offering childbirth care*, *in which midwives take primary professional responsibility for birthing care*”. Within this overall definition, different types of MLBC exist: freestanding (located on a separate site from a hospital obstetric unit), alongside (located on the same site as the obstetric unit but not within it), and onsite (located within the hospital obstetric unit) [13].

Using Covidence software [14], the peer-reviewed articles were organised and screened by a team of six researchers (CH, ST, KH, AN, OB, LP). After the removal of duplicates, the titles and abstracts were reviewed by AN, ST, KH, OB, CH, and articles for full-text review were identified. All authors who did 1^st^ and 2^nd^ reviews, reviewed 23 or more papers. In case of disagreement, the team discussed the issues until a consensus decision was reached. A manual search was performed to identify additional studies from the reference lists of relevant publications.

The full text of identified papers was reviewed to confirm fitting the inclusion criteria and the study characteristics were extracted using a data extraction tool in Excel [15]. We did not undertake a systematic appraisal of the quality or weight of evidence as this scoping review aiming to provide a descriptive overview of the literature [15]. Qualitative data and a narrative summary of quantitative data then were coded against the NOC framework domains and subdomains and analysed deductively [6]. This helped to identify the studies that addressed the enablers and challenges under each domain and sub-domains (Table 3). In the next step, we undertook a thematic analysis of the key findings under each sub-domain (Table 1). Upon completion of each step of data extraction and analysis, the findings were reviewed, discussed, and modified by the team.

**Table 3:** Map of papers addressing the domains of NOC by countries.

				Domain I: Agreement and enabling environment				Domain II: Operational standards				Domain III: Quality, efficiency & responsibility			Domain IV: Learning and adaptation		
	1st Author	Year	Country	Policy environment	Financing: Affordability & service utilisation	Purposeful arrangements: Responsive to the needs	Buy-in & trusting relationships	Referral system	Data driven monitoring & evaluation	Supply & infrastructure	Workforce retention, support, and challenges	Coordination of care and efficiency	Benchmarking: Competence & quality of care	Evidence based clinical guidance	Client centeredness	Flexibility & extending reach	Use of innovative approaches & technology
1	Akhtar	2017	Pakistan		**✓**	**✓**	**✓**	**✓**		**✓**	**✓**	**✓**		**✓**	**✓**	**✓**	**✓**
2	Alonso	2018	Mexico	**✓**	**✓**	**✓**	**✓**				**✓**	**✓**	**✓**	**✓**	**✓**	**✓**	**✓**
3	Alonso	2021	Mexico	**✓**	**✓**	**✓**		**✓**		**✓**				**✓**	**✓**	**✓**	**✓**
4	Anon	2012	South Africa	**✓**	**✓**					**✓**	**✓**	**✓**				**✓**	
5	Anwar	2014	Pakistan		**✓**	**✓**	**✓**				**✓**		**✓**		**✓**		
6	Bogren	2021	India	**✓**			**✓**								**✓**		
7	Caldas Nicacio	2016	Brazil			**✓**	**✓**				**✓**	**✓**					
8	da Silva	2012	Brazil		**✓**			**✓**			**✓**						
9	da Silva	2013	Brazil	**✓**							**✓**			**✓**	**✓**	**✓**	
10	David	2012	India					**✓**			**✓**		**✓**	**✓**			
11	Diba	2019	Indonesia		**✓**			**✓**				**✓**					
12	Dutton	2020	South Africa	**✓**		**✓**	**✓**	**✓**		**✓**	**✓**				**✓**		
13	Erawati	2020	Indonesia	**✓**	**✓**		**✓**	**✓**				**✓**		**✓**			
14	Freitas	2019	Brazil	**✓**		**✓**			**✓**				**✓**	**✓**	**✓**		
15	Hofmeyr	2014	South Africa	**✓**	**✓**			**✓**			**✓**	**✓**	**✓**			**✓**	
16	Horner	2014	South Africa			**✓**	**✓**	**✓**					**✓**	**✓**			
17	Jiang	2018	China								**✓**			**✓**	**✓**		
18	Mahmood	2019	Bangladesh		**✓**	**✓**		**✓**		**✓**	**✓**		**✓**		**✓**	**✓**	
19	Malatji	2020	South Africa								**✓**				**✓**	**✓**	
20	Malesela	2021	South Africa				**✓**			**✓**	**✓**	**✓**	**✓**		**✓**		
21	Moudi	2014	Iran	**✓**	**✓**	**✓**	**✓**	**✓**			**✓**				**✓**	**✓**	
22	Moudi	2016	Iran	**✓**	**✓**	**✓**	**✓**	**✓**	**✓**		**✓**	**✓**	**✓**	**✓**		**✓**	
23	Ngongo	2013	Sierra Leone		**✓**			**✓**		**✓**	**✓**	**✓**	**✓**	**✓**		**✓**	
24	Nunes	2016	Brazil	**✓**	**✓**	**✓**				**✓**	**✓**	**✓**		**✓**	**✓**		
25	Oosthuizen	2017	South Africa		**✓**			**✓**		**✓**			**✓**	**✓**	**✓**	**✓**	
26	Oosthuizen	2019	South Africa								**✓**		**✓**			**✓**	
27	Oosthuizen	2020	South Africa	**✓**						**✓**				**✓**			
28	Progianti	2013	Brazil	**✓**				**✓**			**✓**	**✓**				**✓**	
29	Rodrigues Duarte	2019	Brazil							**✓**				**✓**	**✓**		**✓**
30	Santos	2015	Brazil	**✓**			**✓**							**✓**			
31	Schneck	2012	Brazil				**✓**	**✓**	**✓**			**✓**					
32	Shah	2016	Nepal		**✓**					**✓**						**✓**	
33	Shahinfar	2021	Iran				**✓**							**✓**	**✓**		
34	Shahnaz	2015	Pakistan	**✓**						**✓**	**✓**	**✓**			**✓**	**✓**	**✓**
35	Viana	2012	Brazil													**✓**	
36	Wallace	2019	Bangladesh	**✓**				**✓**		**✓**	**✓**			**✓**		**✓**	
37	Wang	2012	China								**✓**		**✓**		**✓**		
38	Zitha	2020	South Africa			**✓**	**✓**				**✓**				**✓**		
39	Zolala	2019	Iran								**✓**		**✓**				
40	Zulfa	2021	Indonesia								**✓**						

## Results

### Characteristics of included articles

**Fig 1:**
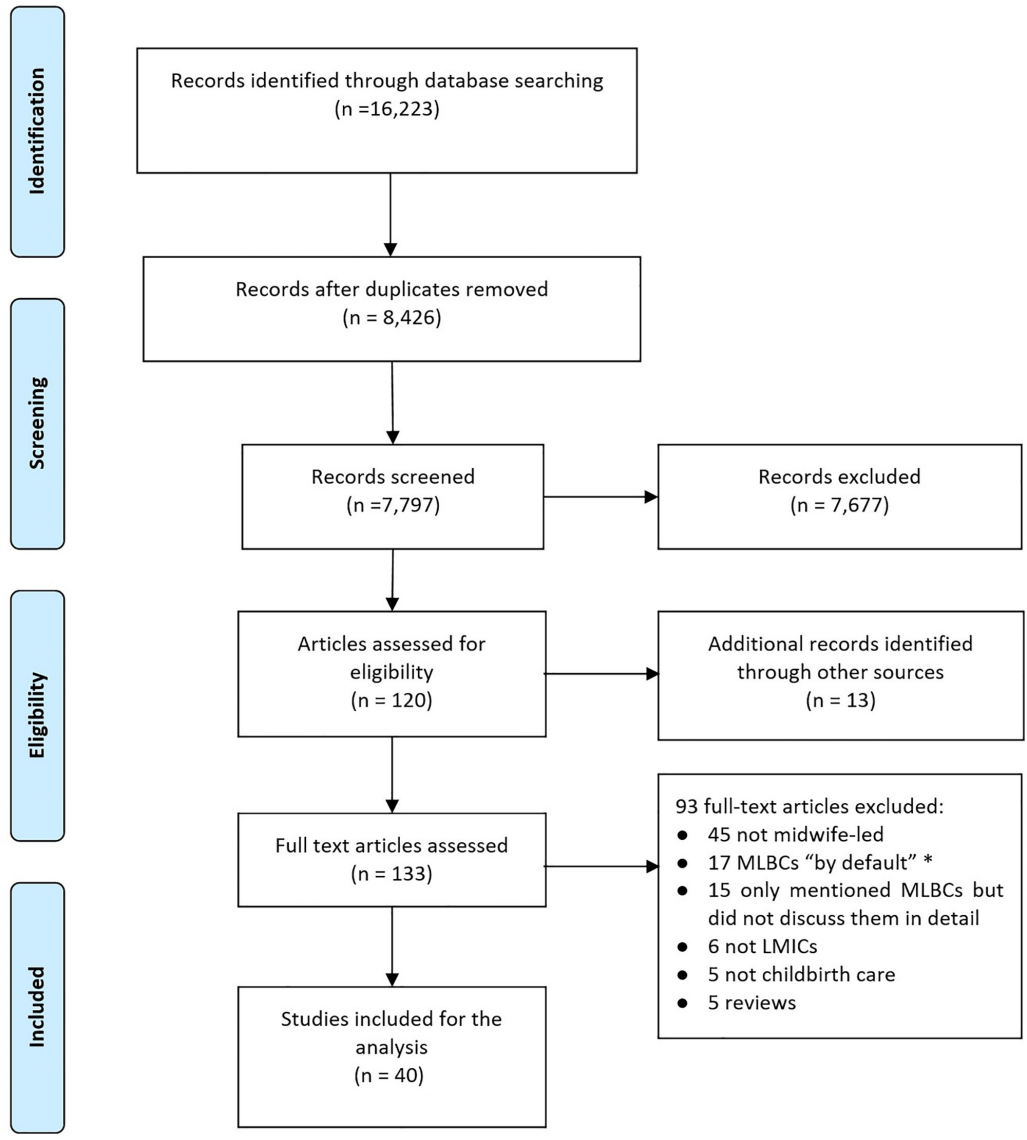
PRISMA diagram of the literature review process.

In total, 16,223 references were identified, of which 8,426 were duplicates. Of the 7,797 remaining references, 7,677 did not meet the inclusion criteria, leaving 120 which had a full-text review, plus 13 additional references located via hand searches (total = 133). Of these 133, 93 were excluded after full-text review, leaving 40 items included (Fig 1).

Of the 40 papers, 25 (63%), were published between 2016 and 2022. There were 36 research studies, three commentaries [16–18] and one conference abstract [19]. These used a variety of methods and study designs. From 36 research papers, three used mixed methods [20–22], 16 were qualitative [5, 23–37], and the remaining 17 used a quantitative design.

The 40 articles represent 12 countries; half were from Brazil (n = 10) and South Africa (n = 10) with smaller numbers from other countries (Table 4).

**Table 4:** List of representing countries by region.

Country	ICM Region	Country income group	Number of papers
**Brazil**	Americas	Upper middle	10
**South Africa**	Africa	Upper middle	10
**Iran**	Eastern Mediterranean	Lower middle	4
**Indonesia**	South-East Asia	Lower middle	3
**Pakistan**	Eastern Mediterranean	Lower middle	3
**Bangladesh**	South-East Asia	Lower middle	2
**China**	Western Pacific	Upper middle	2
**India**	South-East Asia	Lower middle	2
**Mexico**	Americas	Upper middle	2
**Nepal**	South-East Asia	Lower middle	1
**Philippines**	Western Pacific	Lower middle	1
**Sierra Leone**	Africa	Low	1

Note: One paper covered two countries, which is why the numbers sum to 41 rather than 40.

In total, 28 studies covering nine countries (Bangladesh, Brazil, China, Indonesia, Iran, Nepal, Pakistan, Philippines, and South Africa) reported MLBCs in the public sector. Two studies (Pakistan and Indonesia) had MLBCs in a range of sectors including public, private for-profit and private not for profit. Studies from seven countries (Mexico, South Africa, India, Indonesia, Bangladesh, Sierra Leone, and Pakistan) reported on MLBCs that were in the private or not for profit sector (Table 5).

**Table 5:** Sectors in which MLBC operate in 12 countries.

Country	Bangladesh	Brazil	China	India	Indonesia	Iran	Mexico	Nepal	Pakistan	Philippines	Sierra Leone	South Africa
**Public**	X	X	X		X	X		X	X	X		X
**Private for-profit**					X	X			X			
**Private or not-for-profit**	X			X	X		X		X		X	X

Most of the MLBCs were freestanding, that is, located in an entirely separate location from the main health facility. Sixteen studies from nine countries (Bangladesh, Brazil, Indonesia, Iran, Mexico, Pakistan, Philippines, Sierra Leone, South Africa) mentioned freestanding MLBCs. Nine papers from five countries (Pakistan, India, South Africa, China, and Brazil) mentioned one or more onsite MLBCs. Five papers from five countries (South Africa, Indonesia, Brazil, Nepal, and Iran) reported that the country had alongside MLBCs (Table 6).

**Table 6:** Type of MLBC identified in 40 studies from 12 countries.

**Country**	**Freestanding**	**Alongside**	**Onsite**
**Bangladesh**	X		
**Brazil**	X	X	X
**China**			X
**India**			X
**Indonesia**	X	X	
**Iran**	X	X	
**Mexico**	X		
**Nepal**		X	
**Pakistan**	X		X
**Philippines**	X		
**Sierra Leone**	X		
**South Africa**	X	X	X

### Networks of care domains

The majority of papers addressed strengths and challenges related to Domain I (agreement and enabling environment) and Domain II (operational standards) followed by Domain III (Quality, efficiency and responsibility) and IV (learning and adaptation). Subdomains related to workforce, client-centredness and flexibility were addressed in most studies. The subdomains with the least attention included ‘data monitoring and evaluation’ and the ‘use of technology’ (Table 3).

**Fig 2:**
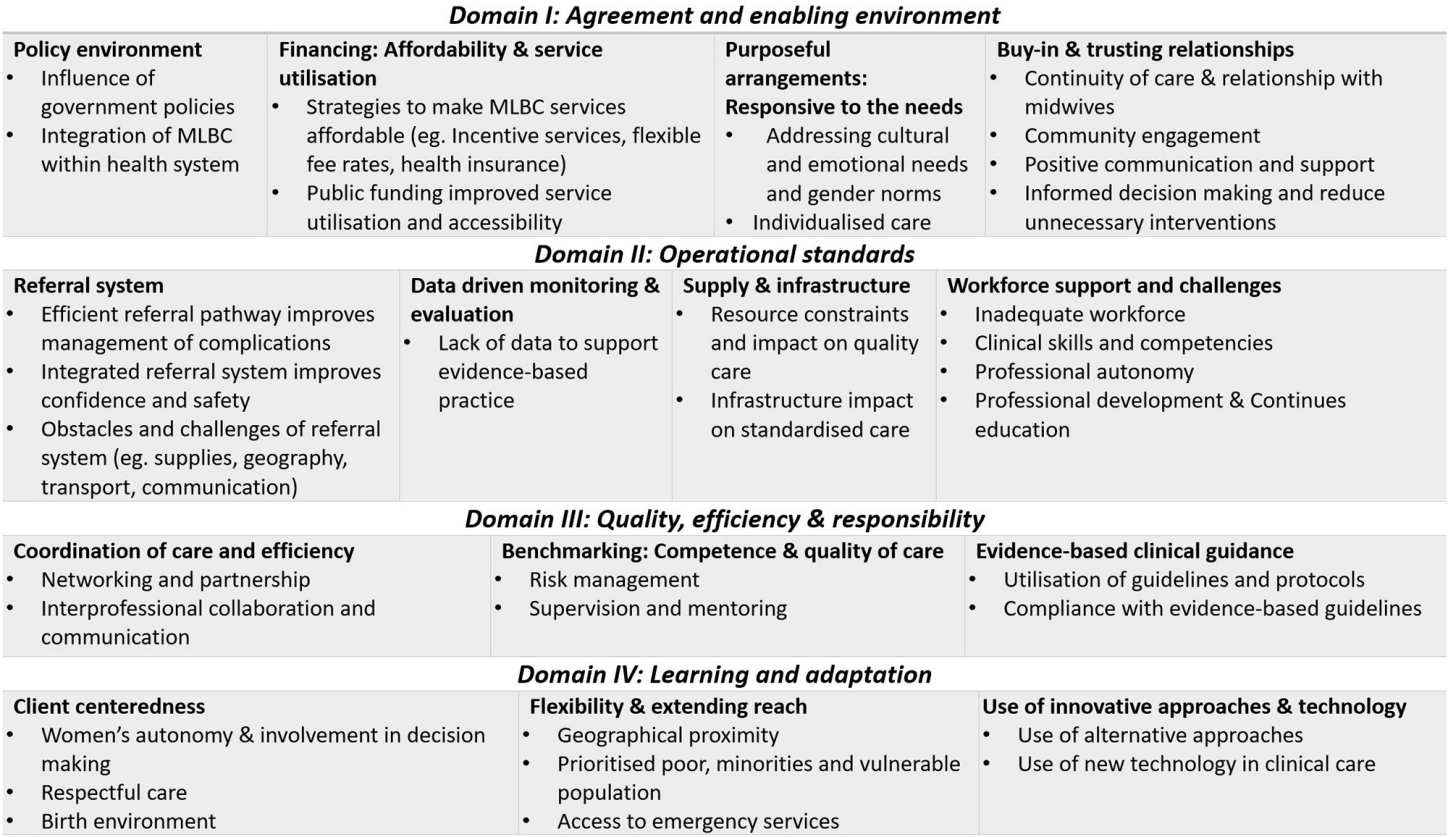
Themes identified under each sub-domains of the NOC framework.

Fig 2 summarises the themes identified under each domain and subdomain, illustrating a range of enablers and challenges to the establishment and operation of MLBCs. Each theme and its subthemes are elaborated in the following section.

### Domain I: Agreement and enabling environment

Domain I includes the policy environment, financing, affordability and service utilisation, purposeful arrangements and buy-in and trusting relationships. The most common issues discussed under this domain related to policy environment, affordability and responsiveness of services and trusting relationship between community and MLBCs. Engagement, agreement, and interaction between all parties involved within the networks of care are essential for MLBC to operate efficiently and ensure high-quality services [6, 8, 9]. Policy agreement and buy-in can be influenced by trusting relationships in an enabling environment with adequate funding. The enablers and challenges under this domain are elaborated in the following sections. One of the unique and positive aspects of supportive policy environment in examples from Brazil, South Africa, Iran and Indonesia indicating that better integration of MLBC within health sector could eventually contribute to improving maternal health indicators and reducing maternal and child mortality [20, 26, 29, 38, 39].

#### Policy environment

A strong policy environment is essential to work collaboratively within a strong NOC. Seventeen papers addressed policy-related issues and factors that enable or restrict MLBCs’ ability to collaborate with other parts of the health system [5, 16, 18, 20, 24–26, 29, 33, 38–44].. Supportive government policies were necessary to enable integration of MLBCs within the health system.

As an example, government policies in Brazil facilitated MLBCs’ integration within the health system, and improved performance of providers by offering care that was fit for purpose and more client-centric [30, 40, 43]. In contrast, in Mexico, midwifery is not well recognised in government policy and the services are underutilised. MLBCs are not well integrated within the health system due to limited linkages between the public and private sectors [24]. Although the Mexican government initiated strategies to increase midwifery services’ utilisation within the public sector, they have not had high level support through an official policy and have therefore had limited impact [33]. In Iran, the government actively discouraged MLBCs and this negatively impacted public opinion and utilisation of MLBC services [20, 41]. Government policy that supports the integration of MLBCs is an essential enabler to the successful implementation of these services [18, 20, 25, 26, 29, 33].

#### Financing: Affordability and service utilisation

Sixteen papers addressed financing including the issues related affordability of MLBC services for women [18, 20, 21, 23, 24, 26, 29, 33, 34, 39, 41, 45–49]. The findings focused on strategies to make MLBC services affordable, such as establishment of a health insurance scheme and incentivised services or fee waivers for maternity services to increase utilization. Communities utilise MLBC services more when the services were either free or subsidised through an enabling environment that included health insurance, incentives, and donor support [18, 20, 24, 26, 33, 39, 41, 45–47].

Lower user fees for MLBC services improved equity of access especially for people from vulnerable populations [18, 20, 33, 34, 46, 48]. Indirect costs, such as transport, food, and family accommodation, were still financial barriers to the utilisation of MLBCs [24, 46, 49]. Providing MLBC services for free is challenging in some contexts as communities may undervalue MLBC services. In Bangladesh, for instance, there was a perception within the community that lower fees must mean lower quality of services at birthing centres and this impacted service utilisation [47].

Relying upon external funds or donor money to subsidise MLBC services, as was reported in Sierra Leone and Mexico, created concern about the sustainability of MLBCs [21, 24]. In Mexico, the only way women can access social welfare is through institutional-based births, such as big hospitals and therefore, MLBCs might not be an option for all women [33].

#### Purposeful arrangements: Responsive to the needs

Being responsive to needs includes enabling meaningful interactions with women and their communities to address their individual, cultural and emotional needs and supporting women and their families to feel safe, confident and included in their care. Thirteen papers addressed these needs [20, 23–25, 28, 29, 32–34, 38, 41, 48, 50].

Being responsive to needs included privacy and respectful and responsive care provided by midwives which was highly appreciated by clients [23, 32]. The provision of high-quality, holistic care within MLBCs increased service utilisation [24, 33. Providing maternity services in a female dominated MLBC environment made women in Brazil and Iran feel safe [20, 28]. The use of non-pharmaceutical interventions and the freedom of choice to make decisions during labour were highlighted as a significant enabler in papers from Brazil and Iran [38, 41].

#### Buy-in and trusting relationship

Fifteen studies addressed issues related to buy-in and trusting relationships [16, 20, 23, 25, 26, 28, 31–34, 37, 41, 43, 50, 51]. Continuity of care, positive communication and informed decision making, non-medicalised approaches, and context-specific needs were critical elements of buy-in and trusting relationships. Approachability and presence of midwives and interaction with a known midwife over time were highlighted as strong features of MLBC by several studies [20, 23, 31, 33, 34, 41]. For instance, women in Pakistan believed that their stress was reduced because they trusted their midwife as they would trust a member of their family [23, 34]. A female-dominated environment within the MLBC was associated with more client satisfaction as they felt it was easy to connect with midwives and develop relationships without gender concerns [28]. This level of engagement was also a means to informed decision making and reducing unnecessary interventions [31, 32, 34, 37].

### Domain II: Operational standards

Domain II highlights the operational standards that are required for a functional NOC including referral, monitoring and evaluation, supplies and infrastructure and a competent workforce.

#### Referral system

The integration of MLBCs within an established referral system improves confidence and a sense of safety among service users. Seventeen articles mentioned issues related to referral systems [5, 20, 21, 23–26, 30, 39–41, 46–48, 50–52]. In all settings, some sort of referral system was in place, but in many cases, it did not function well due to a lack of ambulances and/or equipment and geographical challenges.

In some cases, the geographical location of MLBCs was a challenge to timely management of emergency cases, even if a functional referral system was in place [39, 48, 51]. For example, in South Africa, MLBCs attached to rural health facilities are far from the referral facilities and referral routes are not always functional [22, 42].

In other cases, the lack of integration of the MLBC within the health system makes it difficult to communicate with other facilities to arrange referral [24]. A lack of information and communication about the referral system creates doubt about the safety of care among women [24, 46]. Some countries have developed initiatives to address emergency situations in a timely manner. For instance, MLBC midwives in Sierra Leone were trained in specific emergency life-saving skills to help manage emergencies during ambulance transfer [21].

#### Data driven monitoring and evaluation

A data recording system is important to track MLBCs’ performance and improve quality through regular monitoring. Three studies referred to processes such as organisational audits, monitoring and benchmarking [38, 41, 51]. The studies stated that investment in reliable data collection and interpretation was essential but challenging. For example, in Iran, there was no integrated monitoring system within the MLBCs [41]. In Brazil, after a decade of implementation of MLBC services, the government only recently established a data management system to assess the safety of MLBC care [38, 51].

#### Supply and infrastructure

MLBCs depend on adequate supplies and infrastructure to provide quality care. Fourteen papers highlighted issues related to limited resources and infrastructure and their impact on the standard of care [5, 18, 20, 21, 23–25, 29, 35, 42, 47–49, 53].

Inadequate supplies and infrastructure make it challenging for MLBCs to operate effectively [25, 29]. For instance, in South Africa, poor infrastructure meant a lack of privacy for labouring women [25]. In Bangladesh, social barriers and gender inequality contributed to the lack of supplies and resources: this was reported to be because MLBCs are led by female providers and provide services to women [47]. In a similar way, a lack of medicine and supplies and access to modern technology caused women to avoid MLBCs in Nepal [49]. Both these examples which highlight the gendered impact for the health workforce and the clients show how barriers to supply and infrastructure are multidimensional.

#### Workforce support and challenges

Most studies acknowledged workforce challenges as a major barrier to the effective functioning of MLBCs. Key bottlenecks included midwife shortages, excessive workloads and a lack of clinical competencies, training, and professional autonomy. The studies also emphasised the need for continued professional development for MLBC midwives to maintain clinical skills and competencies [5, 17, 18, 20–23, 25, 27–30, 32–34, 36, 37, 39–41, 44, 45, 52–54].

One of the key workforce enablers was that MLBC midwives were able to practice to their full scope of practice, which boosted the status of midwifery as a profession [23, 28] and led to greater appreciation of the expertise of midwives and how midwifery differs from other health professions [23]. However, this was not the case everywhere. For example, in Brazil, professional and structural barriers prevented MLBC midwives from routinely providing safe care to women [28]. The midwives who worked within these MLBCs had more experience in hospital obstetric units, which sometimes meant they favoured a medicalised model of care rather than a midwifery model [40, 45].

Another example was from Mexico where the use of midwives within MLBCs seems to have promoted the value of midwives and improved the quality of care through collaboration and referrals across health services [24, 33]. Papers from Brazil and Sierra Leone noted that the provision of in-service training provided support, enhanced midwives’ competencies and improved their leadership capabilities [21, 30].

### Domain III: Quality, efficiency and responsibility

Quality, efficiency and responsibility requires coordination of care and efficiency, benchmarking and evidence-based clinical guidance.

#### Coordination of care and efficiency

Coordination of care through communication, networking and partnership and inter-professional collaboration were identified as integral parts of MLBCs. The importance of teamwork in the coordination of care was highlighted in 14 papers [18, 21, 23, 26, 28–30, 33, 37, 39, 41, 46, 51, 53].

In an example from Pakistan, midwives who owned an independent birthing centre reported being well connected, collaborated with other levels of care and provided information about the emergency units, ambulance services, and availability of obstetricians to women and their families. Coordination included providing information to women regarding the steps required if there was an emergency helped women and their families to be knowledgeable and prepared for emergency referrals [23]. However, another study from a private sector MLBC in Pakistan found that midwives had limited opportunities to collaborate with midwives outside their institutions—resulting in poor quality of care due to isolation and lack of diversity of cases in their birthing centre [53].

Some positive examples included Mexico where a network of service providers, governmental public hospitals, and women’s organisations worked together to increase access to the midwifery model of care through collaboration and referral [33] and in Sierra Leone where the specific definition of roles and the clear line of communication, facilitated clinical decision making and coordination. Such coordination helped the system ensure the availability of blood in emergency situations [21]. There were also examples where this element of the NOC was missing. For example, in Indonesia, transport, emergency communication with other facilities and timely referral were not possible due to the lack of partnerships and as a result there were negative impacts on perinatal outcomes [26]. Similarly, in Iran, due to the lack of communication between MLBCs and referral facilities, risk of maternal and neonatal complications arose [20].

#### Benchmarking: Competence and quality of care

The level of competence of providers and community satisfaction with MLBC services, were mentioned as important benchmarks of quality of care in 14 studies [17, 21, 22, 33, 34, 37–39, 41, 44, 47, 48, 50, 52]. These studies suggest the need for improving the competencies of midwives working within MLBCs, with more emphasis on risk management and continuous professional development via in-service training and supportive supervision and mentoring.

The need for capacity building on risk management and emergency obstetric and neonatal care was emphasised by studies from Mexico, South Africa, and Sierra Leone [21, 24, 39]. Supportive supervision and mentorship with further capacity building improved clinical competence and effective risk management [22, 41, 47, 52]. The studies highlighted that experienced and competent midwives add value to the MLBC environment and improve client satisfaction [34, 37].

#### Evidence-based clinical guidance

Eighteen studies indicated the barriers or facilitators for the use of guidelines in delivering evidence-based services [5, 21, 23, 24, 26, 29, 31, 33, 35, 38, 40–43, 50, 52, 54]. In general, the findings relating to the use of guidelines and protocols and the ability of staff to implement and be compliant with evidence-based guidelines, within MLBCs was better than other types of health facilities. However, in almost all settings there was an emphasis on implementing effective clinical guidelines for prevention of complications and unnecessary interventions.

In one example from Brazil, MLBC staff were involved in the development of clinical protocols, and this helped them ensure they were well connected within the higher referral levels [30]. Similarly, MLBCs in Mexico had positive experiences of producing their own guidelines by adapting international guidelines and modifying measures for their unique situation [24].

The incorporation of evidence-based care is a challenge for health services, including MLBCs [43]. For example, an obstacle in implementing guidelines in Brazil came from a lack of commitment to the midwifery model of care, clashes between members of care teams, lack of infrastructure, lack of familiarity of pregnant women and their families with the MLBC model of care, and sometimes staff values and attitudes [29, 35]. Similarly, in South Africa, guidelines provide treatment recommendations and referral criteria, as well as norms and standards with regard to personnel and equipment at clinics. However, compliance with these guidelines is not optimal—hence high-risk births happened in MLBCs even though this went against the guidance [50]. In Mexico, the MLBC is not part of the government structure so they faced difficulties to develop their guidelines, and sometimes it was inappropriate to adapt guidelines from high income countries for use in LMICs [24].

### Domain IV: Learning and adaptation

Domain IV highlighted the value of learning and adaptation including being client centred, being flexible and extending the reach of the services and using up-to-date technology and innovative approaches to care provision.

#### Client centeredness

Different aspects of client centred care such as safety, autonomy and shared decision making were recognised in 20 studies [16, 17, 20, 23–25, 27, 29, 31–35, 37, 38, 40, 41, 47, 48, 53, 54]. An important issue was woman’s autonomy and choice [23, 29, 34, 35, 38, 47]. Respecting women’s preferences brings about a sense of empowerment and safety and engages them in their own care [34, 38, 47]. Giving alternative options to women such as different labour and birth positions [20, 34, 35, 38] and immersion in water [35] promoted client centred care.

Despite restrictive gender norms in some countries, the MLBC environment provided a safe space that maximised women’s privacy and trust in their care providers [23, 33, 34]. For instance, encouraging labour and birth companions, and as well having female care providers, gave a sense of safety and confidence in MLBC services [23, 24, 27, 54]. Other positive aspects were freedom of mobility, eating and interactions with others [20, 23, 34, 41]. In Mexico, women who had experienced domestic violence found the MLBC to be a safe place [33]. Women in Pakistan strongly acknowledged that MLBC midwives were particular about respecting and maintaining women’s privacy [23, 34]. However, midwives in South Africa sometimes found it challenging to respect privacy and make room for implementing policies due to other resource limitations. This sometimes led to verbal abuse, neglect, and lack of routine monitoring, which adversely affected quality of care [25, 27, 32].

Respecting women’s values and culture and continuous interaction with midwives made MLBC services more attractive to women [23, 31, 33, 34, 53]. In Pakistan, Brazil, Iran, Mexico and South Africa, using simple language and offering culturally sensitive care, such as providing services by female providers, were reported as being important aspects of care that were only offered within MLBCs [23, 31, 33, 34, 37, 53]

#### Flexibility and extending reach

Flexibility and attempting new approaches to extend reach can be important for accessibility of care, especially when providing services for underserved communities. Eighteen studies mentioned geographical proximity to the community being served and financial resources as significant factors in access to MLBC services [5, 18–24, 27, 30, 33, 39–41, 47–49, 53]. Bringing MLBC services closer to women improved their trust in and acceptance of services in Pakistan, Iran, Bangladesh, and Nepal [5, 23, 41, 49]. In South Africa, opening of MLBCs alongside the hospitals, created a greater capacity for the hospitals to focus on management of complications, therefore increasing access to emergency obstetric and neonatal services [39]. However, despite the availability of MLBC services in rural areas, some women still could not use the services due to lack of transport, geographical or climate and extreme weather conditions [39, 49, 53].

In some settings, MLBCs were designed to address financial barriers to accessing childbirth care. For instance, in Brazil, the location for the new MLBC was a peripheral area “where a large resident population lacks access to public health services in comparison to those in the better financial condition who live in central neighbourhoods,” and that attracted more women from low socio-economic groups to use the MLBC [30, 40]. In contrast, in Sierra Leone, the MLBC was located in the capital city, therefore, inaccessible to the rural population, and there is an urgent need for better access to skilled childbirth care in rural areas [21]. In Mexico, the government covered 50% of the transport costs for emergency cases [33].

Some studies described how MLBCs were established in locations where they could serve women from specific social and cultural groups who may otherwise find accessing care more challenging, e.g. specific ethnic groups, refugees and survivors of violence [20, 24, 27, 33, 47]. In Mexico, the issue of isolation of Indigenous communities from the mainstream led to distrust of government services. However, the establishment of MLBC provided a safe space for Indigenous women to use MLBC services and recover their trust in health care providers [24, 33].

#### Use of innovative approaches and technology

Innovative approaches and technologies include using mHealth. There was limited information on the use of technology or other innovative approaches within the MLBCs. Five papers from Pakistan, Mexico and Brazil addressed this issue with emphasis on use of non-medicalised complementary methods [23, 24, 33, 35, 53]. Other examples included the use of social media and computer applications [23, 24, 33, 35]. In Brazil, MLBCs offered non-medicalised complementary methods, such as aromatherapy [35]. In Pakistan and Mexico, MLBCs provided alternative methods such as mind diversion therapy, particularly during the intrapartum period for pain relief or minimising the intensity of pain [23, 24].

## Discussion

The aim of this review was to evaluate whether the NOC framework—in light of the literature about MLBCs—can be used to map the challenges, barriers and enablers to the establishment and operation of MLBCs, with a focus on low-to-middle income countries.

The NOC framework is a useful way to analyse an MLBC, either at the point of design and implementation or as part of monitoring and evaluation. In terms of the NOC domains, we found evidence that a lack of supportive policies, leadership, inter-facility collaboration, responsiveness to community needs can be barriers to the establishment and operation of MLBCs, and financing and monitoring need to be addressed.

The effectiveness of a NOC within a health system is the responsibility of all involved stakeholders and all levels of care [8]. Therefore, investment in NOCs with adequate operational standards, infrastructure and equipment is essential for the successful delivery of services and for the sustainability of an intervention such as an MLBC [9]. The leadership capacity of the government is critical for the successful integration and implementation of MLBCs within NOCs. Governments may establish a strong NOC through collaboration with different public and private partners and NGOs to institutionalise MLBC as a priority within their country. However, organising and facilitating such multisectoral policy dialogue on delivery of maternity services should be context-specific [55].

While the NOC framework seems to be useful to map the enablers and identify barriers, one of the challenges in using it is the interrelation and sometimes, overlap, between the four NOC domains. It is not possible to separate out the domains and elements of all domains are needed. For example, the mapping showed that without a strong policy enforcement and adequate competent workforce and supply availability, it is not possible to implement quality care or develop trusting relationships with women or engage the community. Some studies are also more conducive to using the NOC framework although we were able to explore the domains in all study designs. Qualitative studies are often more able to explore issues to do with relationships and collaboration, whereas quantitative studies may provide more numeric data on monitoring and evaluation, benchmarking or measures of quality of care.

Delivering MLBC services for underserved and vulnerable populations requires a client or woman-centred approach that is fit for purpose and fulfils the cultural and individual needs of the community [56, 57]. However, such an approach requires a clear strategy for engagement with the community and promote equitable access to services [8, 58]. This was initiated in some contexts by offering incentives and free services for all women and their families as explored in the NOC analysis. Bringing services closer to users helped to engage them more, especially in rural areas [8].

The integration of monitoring and evaluation, including resource mobilisation, service auditing and budgeting the implementing organisations may adopt or evolve changes based on community needs [7]. In rural Madagascar for instance, adaptation of extensive monitoring and evaluation by use of rigorous research and technology not only ensured the functionality of the NOC and its continuous improvement, but also addressed the financial sustainability issues and how the intervention should be scaled up in future [8].

Fewer studies elaborated on, or discussed, the aspects of the NOC framework that included financing, sustainability and affordability of MLBC services. One key financial challenge within a NOC framework is a shortage of human resources; health workforce limitations and the high turnover of competent providers, leadership transitions as well as managerial changes [12]. Many countries undertook critical steps such as establishment of health insurance schemes, incentives for providers to increase motivation and retention, and waiving fees for maternity services.

### Implications

This scoping review has shown that all elements of the NOC framework are important and must be included for the success and sustainability of MLBCs. The NOC framework could be useful tool when MLBCs are being established or evaluated to highlight the strengths and the deficiencies of individual services or policies. Future implementation and evaluation efforts need to assess the effectiveness of MLBCs on maternal health outcomes and provide evidence on the potential strategies for scale-up.

Strengthening the NOC domains could result in a paradigm shift towards more equitable access to high-quality maternity services. The development of an assessment tool to address gaps and overcome barriers in similar settings may be a useful next step. Future research could use the NOC framework as a way to study the interaction of barriers and enablers and the influence of NOCs on the successful implementation of MLBCs in LMICs.

### Strengths and limitations

To our knowledge, this is one of the few studies that has used a NOC framework to map the current evidence in relation to challenges, barriers and enablers to the establishment and operation of MLBCs. It provides valuable insights for those seeking to scale up this model of care. One of the key strengths of this review is the use of the NOC framework which underlines the importance of the NOC dimensions when establishing any model of care. We recognise however that the NOC domains were our deductive categories. They were not necessarily the categories or themes identified in the included articles and that the results are now being considered from the perspective of a NOC. Despite this, we feel that the NOC provides a useful framework when considering the essential elements of implementing MLBCs. Future studies may choose to use the NOC framework a priori and further determine the utility of this approach.

It is clear that there is a lack of evidence around MLBCs in LMICs other than Brazil and South Africa. Many other LMICs have MLBCs that are not featured in the peer-reviewed literature [59]. The knowledge gained is limited to only a few countries which are unlikely to be typical of all LMICs. It is also notable that over half of the papers included in this study are from upper middle-income countries and that may impact the diversity of data interpretation. Another limitation to this review is the variation in the definition of an MLBC both within and between countries. Identifying relevant themes and assigning the concepts to NOC domains and sub-domains might be a subjective process based on the authors’ reflections, knowledge, and expertise. This review did not analyse individual countries’ services and so we are unable to verify the findings from the papers. In addition, in line with most scoping reviews [15], we did not conduct a quality appraisal of the studies.

We acknowledge that restricting the review to English, French and Spanish will have excluded relevant literature in other languages. The search was restricted to literature published between January 2012 and February 2022, and therefore excluded relevant items published outside of this time period.

## Conclusions

This review brings together available empirical evidence about MLBCs in LMICs using a NOC framework. The NOC framework facilitated the identification of gaps and can eventually be adapted for use as an assessment tool to address gaps and overcome barriers in similar settings or may help to develop strategies for implementing sustainable MLBCs successfully.

## Supporting information

S1 Table Databases and search strategy. (DOCX)

S1 Checklist PRISMA 2020 checklist. (DOCX)
