# Supplementary material for: Exploring networks of care in implementing midwife-led birthing centres in low- and middle-income countries: A scoping review
Source: PLOS Glob Public Health. 2023 May 23;3(5):e0001936. doi: 10.1371/journal.pgph.0001936 (PMC10204965; doi:10.1371/journal.pgph.0001936)
Supplement: S1 Table — (DOCX) [file pgph.0001936.s001.docx]

**S1 Table: Databases and search terms for peer-reviewed literature**

| **Database** | **Search terms** |
| --- | --- |
| CINAHL | AB (midwi* OR sage-femme OR matrona or partera or "enfermera obstétrica") AND AB (unit* OR cent* OR unidad OR "maison de naissance" OR "casa de parto" OR “casa de partos”)  Filters: human; language = English, French or Spanish; publication year 2012-2022; geography = Europe, Continental Europe, Africa, Asia, Middle East, Mexico & central/south America |
| Cochrane Library | Title Abstract Keyword: (midwi* OR sage-femme OR matrona OR partera OR “enfermera obstétrica”) AND (unit* OR cent* OR unidad OR “maison de naissance” OR “casa de parto” OR “casa de partos”)  Filter: publication year 2012-2022; Cochrane reviews |
| EMBASE | ('enfermera obstétrica':ti,ab,kw OR midwi*:ti,ab,kw OR 'sage femme':ti,ab,kw OR matrona:ti,ab,kw OR partera:ti,ab,kw) AND (unit*:ti,ab,kw OR cent*:ti,ab,kw OR unidad:ti,ab,kw OR 'maison de naissance':ti,ab,kw OR 'casa de parto':ti,ab,kw OR 'casa de partos':ti,ab,kw) AND [2012-2022]/py AND ([english]/lim OR [french]/lim OR [spanish]/lim) AND [humans]/lim |
| LILACS | Title, abstract, subject: (midwi* OR sage-femme OR matrona OR partera OR “enfermera obstétrica”) AND (unit* OR cent* OR unidad OR "casa de parto" OR “casa de partos”)  Filters: language English, French or Spanish; publication year 2012-2022 |
| MEDLINE | AB (midwi* OR sage-femme OR matrona or partera or "enfermera obstétrica") AND AB (unit* or cent* or unidad or "maison de naissance" or "casa de parto" or “casa de partos”)  Filters: human; language = English, French or Spanish; year of publication 2012-2022 |
| PubMed | (midwi*[Title/Abstract] OR sage-femme[Title/Abstract] OR matrona[Title/Abstract] OR partera[Title/Abstract] OR "enfermera obstétrica"[Title/Abstract]) AND (unit*[Title/Abstract] OR cent*[Title/Abstract] OR unidad[Title/Abstract] OR "maison de naissance"[Title/Abstract] OR "casa de parto"[Title/Abstract] OR “casa de partos”[Title/Abstract])  Filters: last 10 years; Humans, English/French/Spanish |
| Sabinet | Abstract: (midwife OR sage-femme) AND (unit OR center OR centre OR "maison de naissance")  Filters: 2012-2022 |
| Scopus | TITLE-ABS-KEY ((midwi* OR sage-femme OR matrona OR partera OR "enfermera obstétrica") AND (unit* OR cent* OR unidad OR "maison de naissance" OR  casa de parto" OR “casa de partos”)) AND PUBYEAR > 2011 AND(LIMIT-TO ( LANGUAGE , "English") OR LIMIT-TO (LANGUAGE, "French") OR LIMIT-TO (LANGUAGE, "Spanish"))  Filters: Geography - exclude individual high-income countries |
| Web of Science | (TS=(midwi* OR sage-femme OR matrona or partera or "enfermera obstétrica")) AND TS=(unit* or cent* or "maison de naissance" or "casa de parto")  Filters: language = English, French or Spanish, Geography = exclude individual high-income countries; Year of publication after 2011 |
